# Supplementary material for: Nutritional and Inflammatory Markers Associated with SARS-CoV-2 Infection in the Elderly
Source: Int J Mol Sci. 2024 Jul 15;25(14):7749. doi: 10.3390/ijms25147749 (PMC11277511; doi:10.3390/ijms25147749)
Supplement: Supplementary file 1 [file ijms-25-07749-s001.zip › ijms-3042060-supplementary.pdf]

**Supplementary Table S1.** Primers sequences.

| Primer | Forward | Reverse |
|---|---|---|
| IL6 | 5'-AGACAGCCACTCACCTCTTCAG-3' | 5'-TTCTGCCAGTGCCTCTTTGCTG-3' |
| MAPK14 | 5'-GAGCGTTACCAGAACCTGTCTC-3' | 5'-AGTAACCGCAGTTCTCTGTAGGT-3' |
| NFKB1 | 5'-GCAGCACTACTTCTTGACCACC-3' | 5'-TCTGCTCCTGAGCATTGACGTC-3' |
| TNF | 5'-CTCTTCTGCCTGCTGCACTTTG-3' | 5'-ATGGGCTACAGGCTTGTCACTC-3' |
| VDR | 5'-CGCATCATTGCCATACTGCTGG-3' | 5'-CGCATCATTGCCATACTGCTGG-3' |
| IL1B | 5’-CCACAGACCTTCCAGGAGAATG-3’ | 5’-GTGCAGTTCAGTGATCGTACAGG-3’ |
| CASP3 | 5’-GGAAGCGAATCAATGGACTCTGG-3’ | 5’-GCATCGACATCTGTACCAGACC-3' |
| CXCL8 | 5’-GAGAGTGATTGAGAGTGGACCAC-3’ | 5’-CACAACCCTCTGCACCCAGTTT-3' |
| IFNG | 5’-GAGTGTGGAGACCATCAAGGAAG-3’ | 5’-TGCTTTGCGTTGGACATTCAAGTC-3' |
| CXCL10 | 5’-GGTGAGAAGAGATGTCTGAATCC-3’ | 5’-GTCCATCCTTGGAAGCACTGCA-3' |
| CCL2 | 5’-AGAATCACCAGCAGCAAGTGTCC-3’ | 5’-TCCTGAACCCACTTCTGCTTGG-3' |
| IFNA2 | 5’-TGGGCTGTGATCTGCCTCAAAC-3’ | 5’-CAGCCTTTTGGAACTGGTTGCC-3' |
| IFNB1 | 5’-CTTGGATTCCTACAAAGAAGCAGC-3’ | 5’-TCCTCCTTCTGGAACTGCTGCA-3' |
| OAS1 | 5’-AGGAAAGGTGCTTCCGAGGTAG-3’ | 5’-GGACTGAGGAAGACAACCAGGT-3' |
| MX1 | 5’-GGCTGTTTACCAGACTCCGACA-3’ | 5’-CACAAAGCCTGGCAGCTCTCTA-3' |
| IL10 | 5’-TCTCCGAGATGCCTTCAGCAGA-3’ | 5’-TCAGACAAGGCTTGGCAACCCA-3' |
| IL4 | 5’-CCGTAACAGACATCTTTGCTGCC-3’ | 5’-GAGTGTCCTTCTCATGGTGGCT-3' |
| IL17A | 5’-CGGACTGTGATGGTCAACCTGA-3’ | 5’-GCACTTTGCCTCCCAGATCACA-3' |
| TGFB1 | 5’-TACCTGAACCCGTGTTGCTCTC-3’ | 5’-GTTGCTGAGGTATCGCCAGGAA-3' |
